# Functional and proteomic analysis of *Ceratonova shasta* (Cnidaria: Myxozoa) polar capsules reveals adaptations to parasitism

**DOI:** 10.1038/s41598-017-09955-y

**Published:** 2017-08-21

**Authors:** Gadi Piriatinskiy, Stephen D. Atkinson, Sinwook Park, David Morgenstern, Vera Brekhman, Gilad Yossifon, Jerri L. Bartholomew, Tamar Lotan

**Affiliations:** 1 Marine Biology Department, The Leon H. Charney School of Marine Sciences University of Haifa, Haifa, 31905 Israel; 2 Department of Microbiology, Oregon State University, Nash Hall 226, Corvallis, Oregon, 97331 USA; 3 Faculty of Mechanical Engineering, Technion, Haifa 32000 Israel

## Abstract

Myxozoa is a diverse, speciose group of microscopic parasites, recently placed within the phylum Cnidaria. Myxozoans are highly reduced in size and complexity relative to free-living cnidarians, yet they have retained specialized organelles known as polar capsules, akin to the nematocyst stinging capsules of free-living species. Whereas in free-living cnidarians the stinging capsules are used for prey capture or defense, in myxozoans they have the essential function of initiating the host infection process. To explore the evolutionary adaptation of polar capsules to parasitism, we used as a model organism *Ceratonova shasta*, which causes lethal disease in salmonids. Here, we report the first isolation of *C. shasta* myxospore polar capsules using a tailored dielectrophoresis-based microfluidic chip. Using electron microscopy and functional analysis we demonstrated that *C. shasta* tubules have no openings and are likely used to anchor the spore to the host. Proteomic analysis of *C. shasta* polar capsules suggested that they have retained typical structural and housekeeping proteins found in nematocysts of jellyfish, sea anemones and *Hydra*, but have lost the most important functional group in nematocysts, namely toxins. Our findings support the hypothesis that polar capsules and nematocysts are homologous organelles, which have adapted to their distinct functions.

## Introduction

Myxozoa is a large and diverse group of microscopic endoparasites, which have been shown by morphological and phylogenomic analyses to belong within the phylum Cnidaria^1–7^. With more than 2000 species, myxozoans comprise about 20% of this ancient group, which includes corals, sea anemones, jellyfish and hydrozoans^8, 9^. The life cycle of myxozoan parasites alternates between vertebrate (mostly fish) and invertebrate (mostly annelid) hosts^9–11^. Consisting of only several cells, myxozoans are much reduced in size and tissue complexity compared to free-living cnidarians^6, 12^. Nevertheless, myxozoans retain the phylum-defining stinging organelle, known as cnidocyst or nematocyst, but referred to historically as “polar capsule” in Myxozoa^2, 13–15^.

The nematocyst of free-living cnidarians is likely the most evolutionarily ancient venom delivery apparatus in extant multicellular organisms. These organelles are present in large numbers and operate as a micro-weaponry system capable of entangling and penetrating prey or predator, and injecting toxins at an ultrafast acceleration of more than 5 million *g* ^16–18^. The nematocyst contains a tightly coiled needle-like tubule, which everts explosively during the discharge process, then elongates and penetrates the target within a fraction of a second^16–19^. In free-living cnidarians, the driving force of this firing process is a matrix of large aggregates of poly-γ-glutamate (pγGlu) and metal cations, which are trapped inside the capsule and build the high initial osmotic pressure (150 bars) for the discharge mechanism^20–22^. During tubule elongation the pγGlu is integral in driving tubule release from its moving front^23^.

Myxozoans typically have only two or three polar capsules, which are essential for the initiation of host infection. The fired tubule anchors the spore to the host, in some cases by contracting to achieve closer contact, and subsequently injecting the capsule content^24–26^. The first myxozoan whole-genome data contain genes encoding capsule-specific structural proteins, including minicollagens and nematogalectins^13^; however, little is known of the composition of the polar capsule content, and if it shares any similarity to nematocysts of free-living cnidarians, despite the organelles having different functions. Identification of genes that are expressed specifically in these capsules would facilitate better understanding of myxozoan evolutionary adaptations to parasitism.

Recent proteomic studies performed on various nematocyst types from free-living cnidarians identified tens to hundreds of proteins, including nematocyst-specific ones and toxins^27–33^. Recently, we compared the nematocysts of the sea anemone (*Anemonia viridis*), jellyfish (*Aurelia aurita*), and *Hydra magnipapillata* ^34^, which belong to three basal cnidarian lineages that diverged >600 Ma. We found that although only a few proteins were common to all three organisms, many protein domains were shared. This suggested that both common proteomic functions and dynamically evolving species-specific protein profiles are present^34^. However, whereas free-living cnidarians use their capsules to paralyze or deter their targets, myxozoans use theirs to infect a host, a very different task that may involve specific adaptations. To test this hypothesis, we analyzed the content of myxozoan polar capsules and compared our findings with nematocysts of free-living cnidarians.

**Figure 1:**
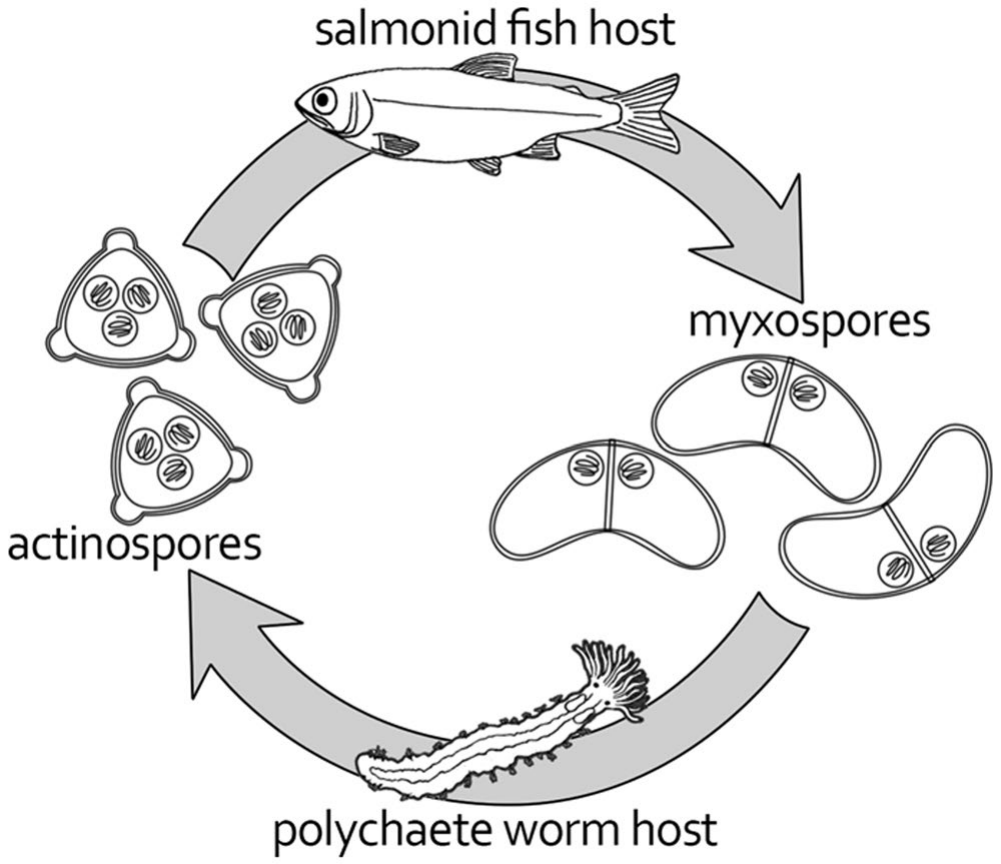
Life cycle of *Ceratonova shasta* showing alternation between salmonid fish and polychaete worm hosts, infected by actinospore or myxospore, respectively. Myxospores have two polar capsules, and actinospores have three.

Our model myxozoan *Ceratonova shasta* infects salmon and trout causing enteronecrosis, a lethal disease characterized by intestinal hemorrhage and necrosis. *C. shasta* is endemic to the Pacific Northwest of North America^35^ and is an economically important pathogen^36–38^. It is one of a few myxozoans whose life cycles have been resolved, shown to involve two morphologically distinct waterborne stages: the actinospore, which infects the salmonid and contains three polar capsules, and the myxospore that infects the freshwater polychaete *Manayunkia* sp. and contains two polar capsules^11^ (Fig. 1).

Here, we report the first isolation of polar capsules from *C. shasta* myxospores using a “lab on a chip” approach^39^, with subsequent proteomic profiling and functional analyses of the purified capsules. Our results provide intriguing insights into the evolutionary adaptation of cnidarians to parasitism.

## Results

### Isolation of polar capsules

**Figure 2:**
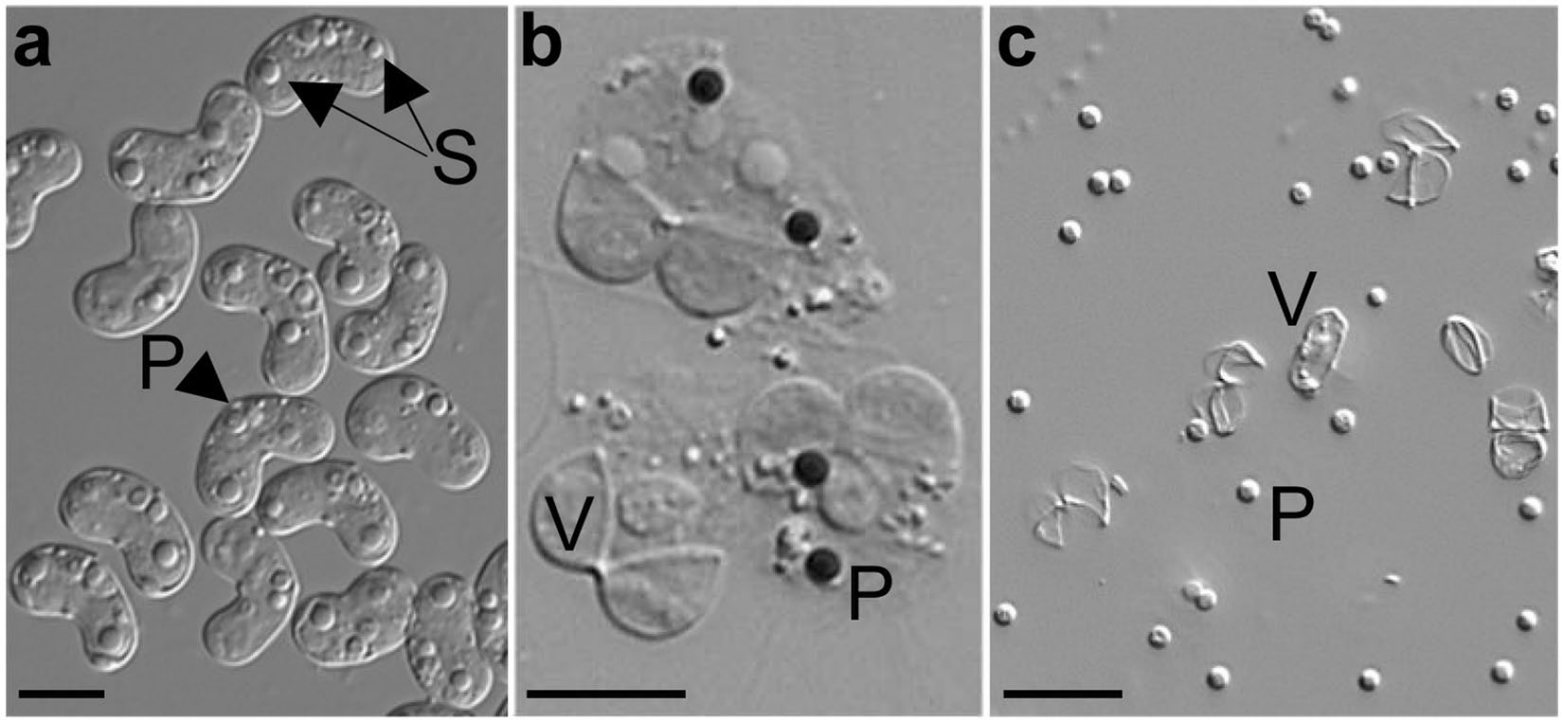
Isolation of polar capsules from whole myxospores. (**a**) Intact *C. shasta* myxospores with two polar capsules (P) and a binucleate sporoplasm (S). (**b**) Myxospores after valve cells (V) have been opened by SDS treatment, and polar capsules (P) stained dark with Toluidine blue. Note that polar capsules and valves are still held together with the spilled content of the spore. (**c**) Dissociated polar capsules and valves following enzymatic digestion. Scale bars = 5 μm.

In comparison to the thousands to millions of capsules found in free-living cnidarians, the *C. shasta* myxospore contains only two polar capsules (r = 0.9 μm), which are tightly embedded within two valves (Fig. 2a), considerably limiting the availability of study materials. To address this scarcity, we first experimented with methods for capsule isolation. We determined that treatment with SDS ‘unstitched’ the suture between the spore’s two valve cells, undoing the spore’s structure. However, the polar capsules remained attached to the inner content of the spore, and gentle proteolytic treatment was required to release them, which resulted in a mixture of unattached polar capsules and valves (Fig. 2b,c).

**Figure 3:**
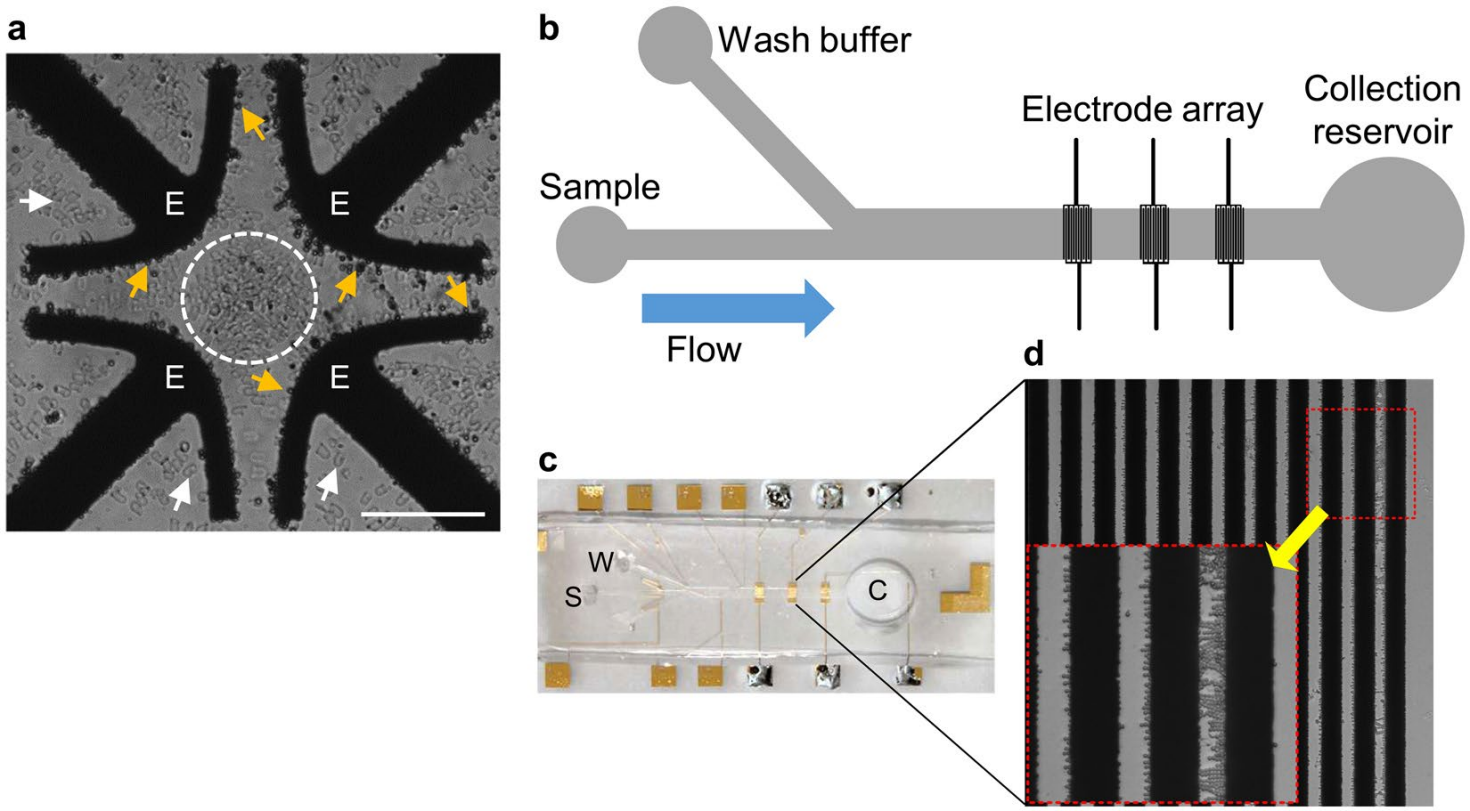
DEP trapping and characterization of polar capsules. (**a**) Light microscope image of a mixture of capsules and valves within a quadrupole electrode array chip, used for DEP characterization, at 10Vpp and 2 MHz. Note that the capsules (yellow arrows) are trapped (i.e. pDEP) on the edge of the electrodes (E), whereas valves (white dashed circle and white arrowheads) are repelled (i.e. nDEP) from the electrodes. Scale bar = 100 μm. (**b**) Schematic description of the DEP-based microfluidic chip, which consists of a main channel with embedded electrode arrays. (**c**) A photo of the DEP-based chip, showing the sample loading (S), wash buffer (W) channels, and the collection reservoir (C). (**d**) Magnified view of the electrode array with trapped polar capsules. Inset (red box) shows magnified electrode and capsules. The entire separation process is shown in Supplementary Video 1.

The low number of capsules and their relative small size rendered capsule isolation by regular biochemical methods inefficient. To overcome this obstacle we used a dielectrophoretic “lab on a chip” device for separation of capsule sub-populations based on their unique dielectric properties (DEP) and consequent unique DEP response^39^. Initially, we tested the dielectric properties of the capsules using a quadrupolar electrode array to identify different DEP behaviors between polar capsules and valves (Fig. 3a). The measured crossover frequency of both polar capsules and valves were ~6 ± 0.5 MHz and ~0.75 ± 0.15 MHz, respectively. At 5 MHz, the valves were repelled from the field maxima at the electrode edge (i.e. experiencing negative DEP (nDEP)), whereas the capsules were strongly attracted to the edge as a result of positive DEP (pDEP) (Fig. 3a). Taking advantage of these distinct DEP behaviors, we designed a DEP-based microfluidic chip, in which the capsules were attracted to the electrodes (pDEP), while the repelled valves (nDEP) passed over the electrode array (Fig. 3b–d and Supplementary Video 1). Using this tailored DEP-chip platform, we successfully isolated sufficient purified polar capsules for the proteomic analysis. This is the first reported isolation of polar capsules using DEP.

### Functional analysis of the polar capsule

**Figure 4:**
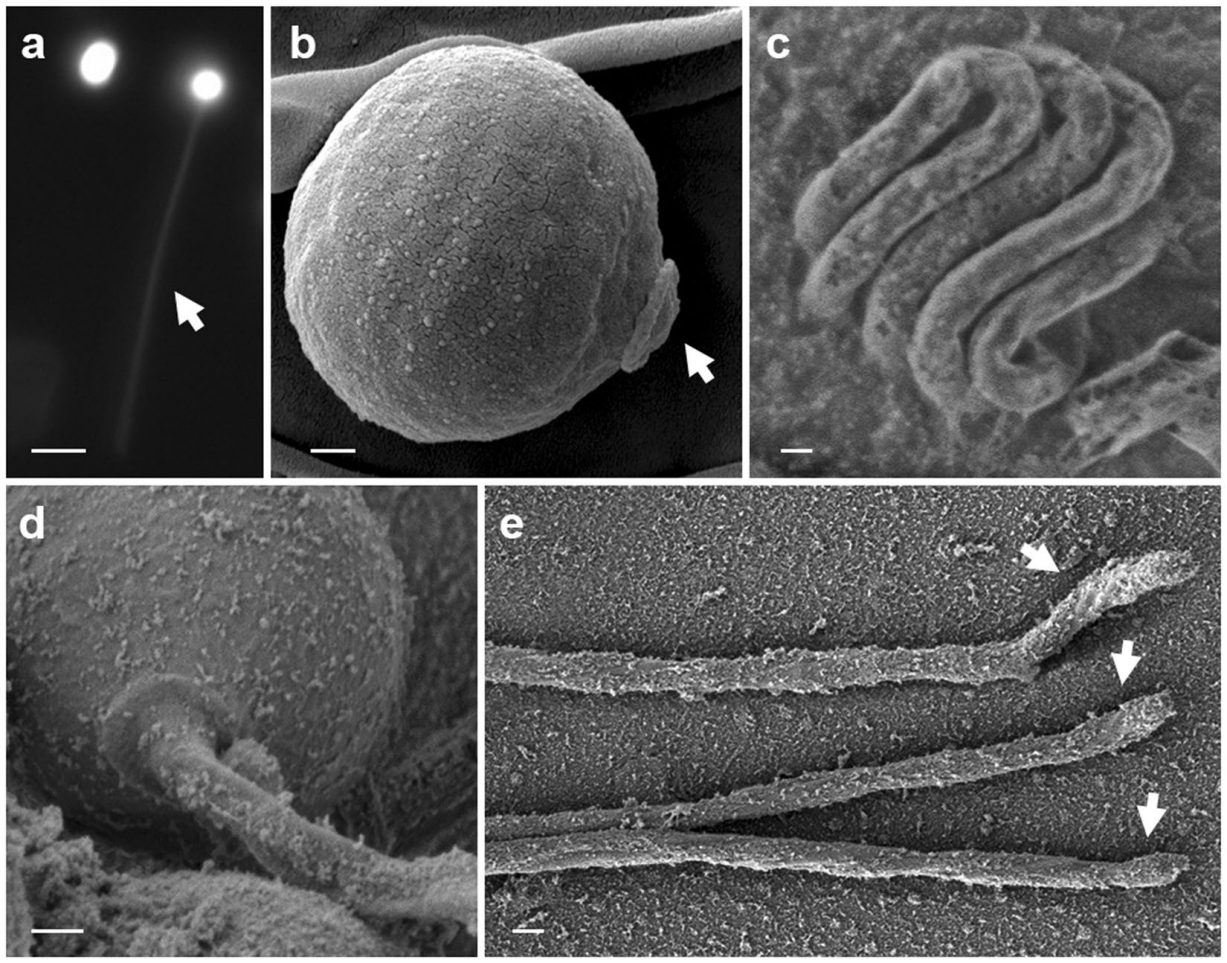
Light microscope and SEM images of polar capsules and tubules. (**a**) Intact and activated polar capsules, pre-stained with Acridine orange. The arrow marks the florescence-stained tubule of the activated polar capsule; note that the activated capsules retain the dye. Bar = 5 μm. (**b**) SEM image of an unfired capsule. The operculum (arrow) is distinct. (**c**) Cryo-SEM image of the inverted, highly-packed tubule folded within an intact capsule. (**d**) Activated capsule with the everted tubule. (**e**) Released tubules with hooked ends (arrowed). Scale bars (**b–e**) 200 nm.

We tested if the polar capsules of *C. shasta* can function as an injection device, similar to nematocysts of free-living cnidarian as well as some *Myxobolus* species that were shown to have open tubules^26, 40, 41^. When dye-labelled polar capsules were activated, the dye spread into the tubule, but no dye release was observed and the capsule remained stained (Fig. 4a). This suggested that polar tubules had no opening and that the dye was trapped inside the activated capsules. SEM analysis of capsule and tubule demonstrated the initial organization of the tubule within the capsule and showed that the released inverted tubule had no spines, barbs or opening (Fig. 4). We found that the tubule ended in a hook-like structure, which might assist the tubule in holding on to the host. These results indicate that *C. shasta* polar capsules function mainly for anchoring the host, and do not inject the capsule content (putative toxins).

### Proteomic analysis

**Figure 5:**
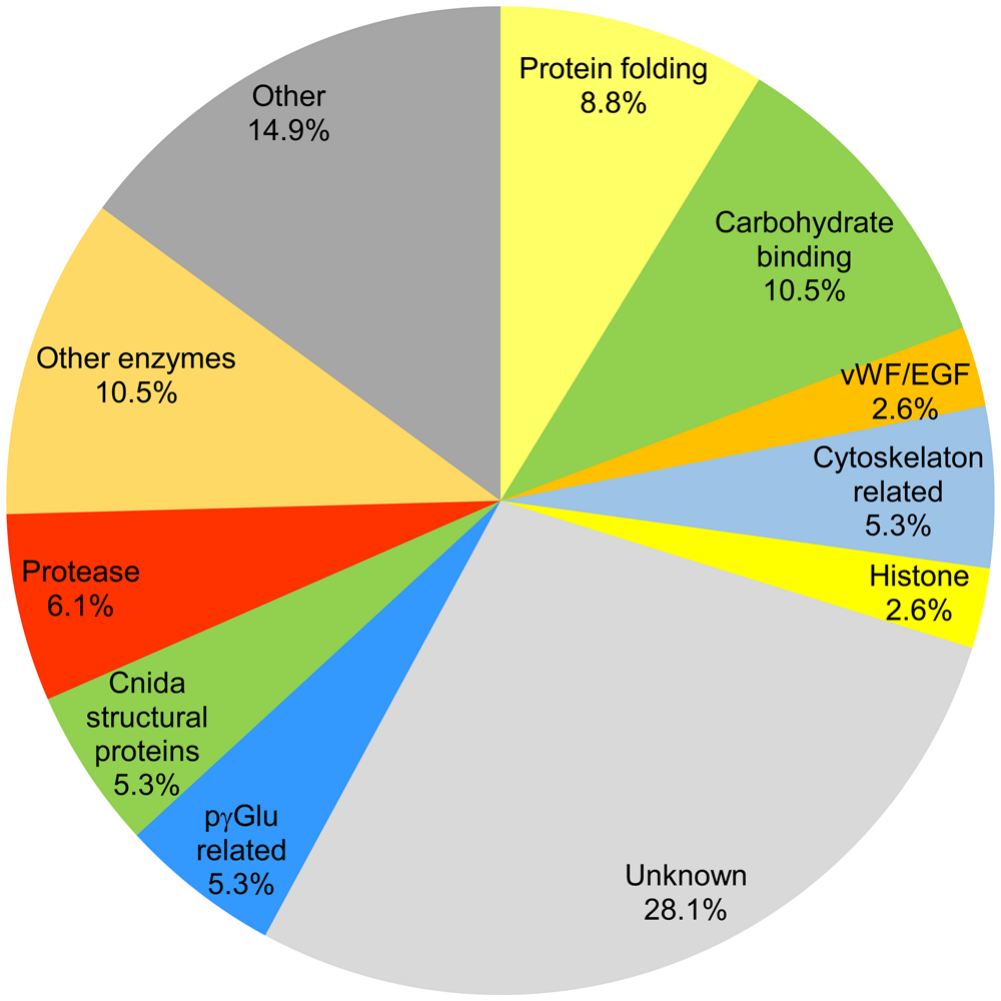
Main protein groups in *C. shasta* polar capsules identified by proteomic analysis. The polar capsule proteomic profile was divided to functional groups based on annotations and protein domain characterization and the percentage of each group from the 112 identified proteins is shown. For the full list, see Supplementary Table S1.

We analyzed proteomes of isolated polar capsules from three biological replicates, each corresponding to a different batch of laboratory-infected fish. Analysis focused on proteins identified with high confidence in at least two of the three replicates. After determining that activated capsules did not inject their content, we analyzed the whole extract of the polar capsules (Fig. 5). We identified 112 proteins, which we divided into subgroups according to their annotation and InterPro (IPR) domains (Fig. 5). The percentages provided hereafter refer to the number of proteins out of the 112 identified. The complete list of proteins, including annotation and the Byonic searches, can be viewed in Supplementary Table S1.

### Polar capsule proteomic profile

Our initial search for phylum-specific capsule structural proteins identified two types: proteins from the nematogalectin family, which are typically found in the tubule; and minicollagens, which comprise the capsule envelope and tubule of nematocysts. Phylogenetic analysis of the three identified nematogalectins in our study revealed their relation to types A, C and R proteins found in myxozoans (Supplementary Fig. 1) and free-living Cnidaria. One type 3 minicollagen was identified in our proteomic analysis and two more type 2 associated minicollagens were found only in one biological sample. These proteins contained characteristic signal peptides and, since they are homologous to nematocyst-specific structural proteins, we considered these valid myxozoan capsule proteins (Supplementary Fig. 2). However, type 1 minicollagen was not found in either our proteomic analysis or in the *C. shasta* transcriptomic data.

We identified a group of proteins related to carbohydrate binding, which had domains including lectin binding, WSC, galactose-binding, and reciprocal BLAST indicated that some of these were similar to uncharacterized proteins of the myxozoan *Thelohanellus kitauei* ^42^ (Supplementary Table 1). Interestingly, several proteins had high similarity to nb012a, a specific *Hydra* protein expressed only in isorhiza type capsules, which is the most simple type, and in desmonemes that have tubules with no opening and are used for prey entanglement^43^. Proteins related to matrix interaction, with EGF-like and von Willebrand factor (vWA) domains, were found also. In the category of protein folding, several proteins that serve as chaperones, such as HSP70, or for protein conformation, such as various isomerases, were found. Additionally, enzymes associated with pγGlu biosynthesis, including γ-glutamyl transpeptidase and glutamine γ-glutamyltransferase were identified. The protease category contained mainly proteins that are related to proprotein processing, such as PC3-like endoproteases, and proteins with metalloprotease domains, which may be used for the maturation of the polar capsules proteins. We identified one putative protease inhibitor belonging to peptidase inhibitor 16, which contained the conserved cap domain and C-terminal extensions of chitin-binding domain. However, the protease inhibitory function of this subfamily is not yet well characterized in mammals or in cnidarians^44^.

The remaining protein groups contained histones, carbonic anhydrases and housekeeping proteins associated with redox balance, ribosomes and cytoskeleton. In addition to proteins of known functions, we identified a group of orphan proteins with no significant homologue in myxozoan or cnidarian databases, which we suggest are novel *C. shasta* polar capsule proteins. Several of these were cysteine-rich and contained short proline-rich stretches, which are found also in minicollagens, suggesting a similar structural function.

### Functional domain comparison

**Figure 6:**
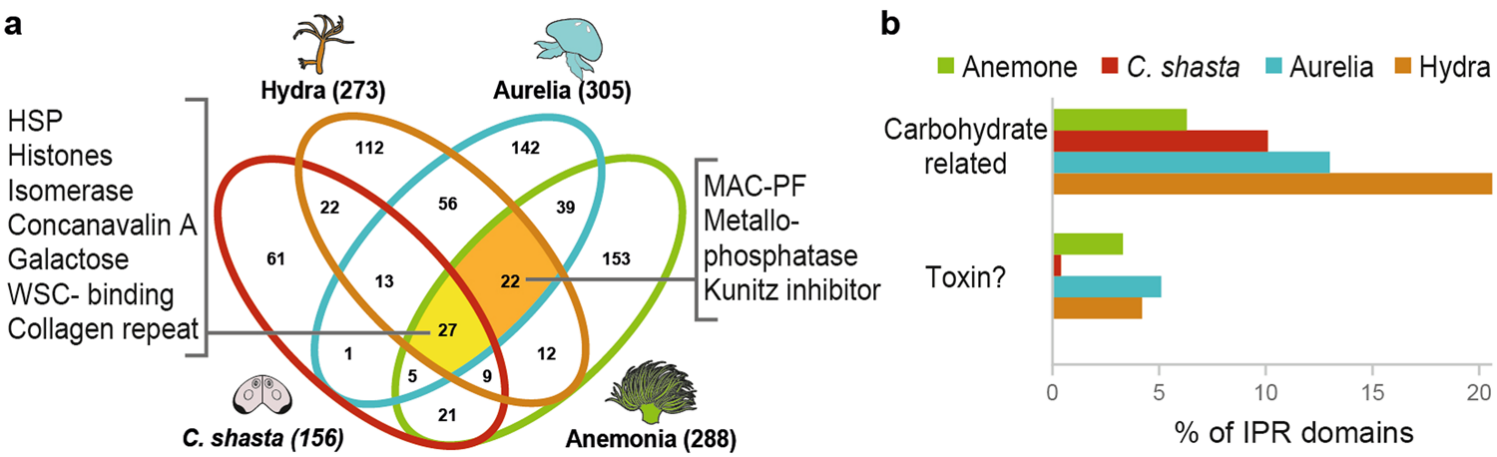
Comparison between capsule proteomic domains of *C. shasta* and free-living cnidarians. (**a**) Venn diagram showing the number of InterPro (IPR) domains shared among the proteomes of *C. shasta*, *Hydra magnipapillata*, *Aurelia aurita* and *Anemonia virdis* capsules. Identified domains for each organism are shown in the brackets. The highlighted sectors contain domains identified in all four organisms (yellow) or only in free-living cnidarians (orange). (**b**) Comparison of the percentages of IPR putative toxins and carbohydrate-related domains in the proteome of *C. shasta* capsules and in the nematocysts of free-living cnidarians. For the full list of identified domains, see Supplementary Table S2.

Previously, we showed that 49 protein domains were shared among the nematocysts of anemone, jellyfish and hydra, representing enriched functions in these organisms^34^. Therefore, to assess the functional similarity between *C. shasta* polar capsules and nematocysts of free-living cnidarians, we compared their protein domains (Fig. 6a). We identified only 27 domains that were shared among free-living Cnidaria and Myxozoa. These included structural domains, HSP, carbohydrate binding, von Willebrand factor, ShK, histones and others (Supplementary Table 2). Intriguingly, among the domains that were shared with the free-living species, but not with *C. shasta*, were ones related to toxins, such as membrane attack complex component/perforin (MAC-PF) domain, metallopeptidase, and the proteinase inhibitor I2 Kunitz domain. Most of the other domains in that group belonged to functional categories that were also found in *C. shasta*.

The absence of these shared toxin domains in the *C. shasta* proteome, prompted us to search specifically for putative toxin domains in the polar capsule proteome. For comparison, we also search for these domains in the protein data from nematocysts of free-living cnidarians, and as a control, we compared InterPro carbohydrate-related domains in all organisms (Fig. 6b). We found that the toxin and carbohydrate domains consisted of 3–5% and 6–20%, respectively, of the IPR domains in free-living Cnidaria. In *C. shasta* polar capsules, carbohydrate domains comprised 10% of the IPR, well within the range of free-living cnidarians. Yet, surprisingly, toxin domains comprised only 0.4%, and included only one ShK domain. However, this ShK domain was in an unknown large protein, with no signal peptide or predicted K^+^ channel function and, therefore, probably does not function as a toxin. Thus, we conclude that *C. shasta* polar capsules do not contain known toxins.

## Discussion

Myxozoa diverged from free-living cnidarians in the Cambrian era, some 500 million years ago^45^, to become a group of microscopic parasitic organisms. During that transition, they lost many genes characteristic of multicellularity, but retained genes associated with the structure of the stinging capsule, which is characteristic of the phylum Cnidaria^6^. Myxozoan polar capsules and the nematocysts of free-living cnidarians were hypothesized to be homologous organelles, though their functions and targets are very different. In the present study, we tested this hypothesis by characterizing polar capsules of the myxozoan *C. shasta*, using proteomic profiling, and structural and functional analyses, and compared our findings to free-living species. This is the first proteomic analysis of Myxozoa and, specifically, of polar capsules.

To overcome the challenge of isolating the polar capsules, we used a DEP-chip platform, which allowed separation and sorting of particles based on their dielectric properties^46^. This approach was preferred over common biochemical methods such as centrifugation or Percol gradients because it yielded relatively large numbers of intact capsules, while offering high controllability and ease of operation. DEP-chip has been applied widely in biomedical research, bacteria detection and cell separation and its potential for jellyfish nematocyst characterization has recently been shown^46–49^; but, it had not been used previously for cnidarian fractionations. We demonstrated that polar capsules had a distinct pDEP response in specific frequency ranges, which enabled their successful isolation and concentration. Given that different particles (organelles) have different DEP characteristics, we anticipate that a DEP-chip could be used for isolation of different types of nematocysts from the same tissue (a feature of free-living Cnidaria), and thereby has the potential for exploring functional specificity of a wide range of nematocysts and polar capsules.

The proteomic profile of the *C. shasta* polar capsule indicated that it is fundamentally similar to nematocysts of free-living cnidarians. We determined that several classes of proteins were conserved in polar capsules: structural proteins, such as minicollagens and nematogalectins; proteins related to protein folding and processing; and nematocyst-specific housekeeping proteins. About 13% of the identified proteins contained carbohydrate binding and matrix interaction domains, which may be signatures of processes known in free-living Cnidaria, including capsule synthesis, which involves secretion from the Golgi apparatus, and tubule synthesis and packing, which involves glycosaminoglycans^50–52^. Moreover, these domains may interact with the host matrix during tubule firing and host anchoring.

The osmotic machinery that drives tubule ejection in free-living cnidarians is based on a unique anionic matrix of pγGlu^16, 20^. Cnidarians are the only known metazoans that can synthesize this γ polymer^21, 53^, although the exact biosynthesis pathway is still unknown. The pγGlu-related enzymes that we identified in *C. shasta* polar capsules, are found also in *Hydra* nematocysts^27, 54^, which may indicate of their role in pγGlu synthesis. Taken together with our finding that there is intense cationic dye staining of the capsule content, we propose that the same principle function of tubule firing is shared between capsules in parasitic and free-living species. However, *C. shasta* polar capsules belong to the simplest group of nematocysts, having no spines or barbs on their tubule and, as we have shown, no openings in the tubule wall or distal end. Thus *C. shasta* capsules appear incapable of injecting the capsule content. This raises the possibility that unreleased high pressure persists inside the capsule and may provide additional rigidity to the elongated tubule, keeping the spore anchored to the host. Interestingly, a specific type of nematocyst in Hydrozoa, the desmoneme, also has no opening and functions to anchor prey by coiling around its bristles. After firing, the tubule of *C. shasta* capsules is straight, except for the structure of the tubule tip, which is hook-like and may facilitate anchoring to the host.

In free-living cnidarians, toxins such as neurotoxins, cytolysins, and metalloproteases are an important part of the nematocyst proteome^34, 55, 56^. Significantly, we did not find any homologous toxins in the proteome of the *C. shasta* polar capsule. These data suggest that these genes/functions have been lost as part of simplification that occurred during the transition from a free-living organism to a microscopic parasite for the Myxozoa as a whole^6^ or, alternatively, be a secondary loss during adaptations in more recent ancestors of *C. shasta* to its particular hosts, combined with structural changes such as closure of the tubule tip. Recently, we showed that many specific toxins found in the three cnidarian lineages Anthozoa, Scyphozoa and Hydrozoa are unique to each of those lineages, having evolved after their diversification^34^. Thus, we hypothesize that similar specification has occurred and is ongoing in Myxozoa, and probably among myxozoan lineages, to generate diverse toxins that may be difficult to recognize. Interestingly, putative myxozoan toxin proteins were identified in transcriptomic analysis of *Myxobolus pendula* ^57^; however, additional studies are needed to verify their function and spatial association with polar capsules. We are currently searching for toxins in the proteomes of other *Myxobolus* species that we have shown to have porous or open tubules and can release their capsule content^26^. We consider it likely that presence of toxins will correlate with open or porous tubule structure, and the corollary for species with closed tubules. Further, we propose that free-living species that possess capsules with closed tubules that cannot inject the capsule content, such as desmonemes in *Hydra*, will not have toxins in their proteomes.

In summary, our findings with *C. shasta* illustrate evolutionary adaptations of polar capsules and tubules as part of an organismal shift from free-living to parasitic. Data from the *C. shasta* proteome and polar tubule structure show that while the basic protein content and the osmotic machinery needed for the explosive function of the polar capsules have been retained, toxins and tubule injection capabilities have been lost.

## Methods

### Myxospore isolation

*Ceratonova shasta* myxospores were obtained from fish that had been infected by exposure to parasite actinospores from our cultures of infected polychaete worms. Fish were held at 14 °C until gross clinical signs of infection (i.e., swollen belly, distended vent, slow swimming) were observed, typically around 30 d post-exposure. Fish were euthanized by bath overdose of tricaine methanesulfonate (MS222) and dissected. Most of the intestine was removed and myxospores were purified using our published methods^58^. Briefly, tissue was macerated and shaken in ~30 mL phosphate buffered saline (PBS), then filtered through a 70 µm mesh cell sieve. Filtrate was pelleted twice by centrifugation and re-suspension in fresh PBS. The crude spore pellet was purified on a Percol gradient, rinsed several times with distilled water to remove Percol, then examined microscopically to verify purity, before drying in a SpeedVac (Savant). All experiments performed using live animals were in accordance with state and federal regulations, and were pre-approved by the Oregon State University, Institutional Animal Care and Use Committee (IACUC). Fish were exposed to the pathogen, monitored and euthanized in accordance with OSU IACUC Animal Care and Use Protocol #4666.

### Polar capsule extraction

To break open the rigid spore structure, the dry pellet was dissolved in 1% sodium dodecyl sulfate (SDS) for 60 min at room temperature while shaking. SDS was washed out with 0.1% Tween 20 by centrifugation at 1000 RCF for 8 min. The resulting pellet was then re-suspended in phosphate buffer containing 1% subtilopeptidase A (Sigma) for 60 min at room temperature. The final preparation was washed twice with 0.05% Tween 20, centrifuged at 1000 RCF for 8 min and the pellet was re-suspended in 0.05% Tween 20. The final sample, containing a mixture of loose intact polar capsules and valves, was stored at 4 °C until further use.

### Polar capsule dye loading and activation

Polar capsules were loaded with 0.01% Toluidine blue or Acridine orange (Sigma) by incubating the spores for 5 min with the dye, followed by DDW wash. Polar capsule activation was induced by 0.1 M NaOH^26^.

### Scanning electron microscopy

Polar capsules were attached to 0.01% poly-D-lysine coated slides, and activated by 0.1 M NaOH. Thereafter, the samples were fixed in 2% glutaraldehyde and 1% paraformaldehyde in 0.2 M sodium cacodylate buffer (pH 7.4) for 2 h at 4 °C, then washed in 0.1 M cacodylate buffer and post-fixed in 1% osmium tetroxide with the same buffer for 30 min at room temperature. Samples were dehydrated in an ascending ethanol series up to 100%, transferred to 100% acetone, air dried, coated by either gold or carbon, and examined using a Zeiss Sigma HD scanning electron microscope (SEM).

### Cryogenic scanning electron microscopy

Cryogenic scanning electron microscopy (cryo-SEM) was performed with a Zeiss Ultra Plus high-resolution SEM, equipped with a Schottky field emission gun and a BalTec VCT100 cold-stage maintained below −145 °C^59, 60^. Specimens were examined at very low acceleration voltage from 1 to 2 kV and at short working distances of 3−5 mm. Both in-the-column (InLens) and Everhart-Thornley (SE2) secondary electron detectors were used. A drop containing the isolated capsules was sandwiched between two gold planchettes and positioned inside special tweezers. The tweezers were then rapidly plunged into liquid ethane at its freezing point (−183 °C), and the sandwich was inserted into a liquid-nitrogen pre-cooled in a “sample-table”. The sample table was then transferred into a BAF060 freeze-fracture system (BalTec AG, Liechtenstein) and split open. The fractured specimens were transferred under vacuum at cryogenic temperature by a BalTec VCT100 shuttle, pre-cooled with liquid nitrogen, to the pre-cooled stage of the HR-SEM.

### A quadrupolar electrode array for dielectrophoretic characterization

A quadrupolar electrode array with 100 μm gaps between the electrodes was fabricated using standard photolithography techniques. Specifically, layers of Au/Cr (200 nm/30 nm in thickness) were evaporated onto a glass substrate and patterned using standard photolithography and lift-off techniques. A flexible silicon chamber of 1 mm in both diameter and depth (Grace BioLabs) was attached to the substrate. The device was used for dielectrophoretic characterization of the capsules and to determine the cross-over frequency (COF) corresponding to the transition from positive dielectrophoresis (pDEP) to negative dielectrophoresis (nDEP) behavior and vice versa^61^.

### Dielectrophoresis-based microfluidic chip for capsule separation

The microfluidic device for DEP separation (DEP-chip) of the capsules consisted of a fabricated polydimethylsiloxane (PDMS) microchannel attached to a glass substrate with three embedded interdigitated electrode arrays. The PDMS microchannel had a main channel (20 mm long, 1 mm wide and 25 µm deep) connecting two sub-microchannels (each 400 μm wide), which enabled the introduction of different solutions (samples and wash buffer solution). Three sets of interdigitated electrodes (32 μm wide with 18 μm gaps) were used to generate multiple zones of electric field gradients over a large area, and to filter different particles by nDEP and pDEP under pressure-driven flow. An automated syringe pump (KDS Legato 200 series, KD Scientific) was used to control flow rates of sample and wash solutions introduced through the two sub-channels.

All solutions contained 0.05% Tween 20 to prevent spore components adhering to the surface of the electrodes. The channel was first filled with wash solution, then the sample was introduced at a low flow rate of 0.8 µL/min. During sample introduction, an alternating current electrical field (4 Vpp, 5 MHz) was generated by a signal waveform generator (33220 A, Agilent) to trap polar capsules by pDEP, whereas the valves were repelled from the electrodes due to their nDEP behavior. After trapping, the sample flow was halted, and wash solution at a flow rate of 1 µL/min was used to empty the micro-channels of residual valves. Then, the trapped capsules were released from the electrodes by turning the voltage off, and stored at −80 °C before further analysis. The entire isolation process was monitored with an Andor Neo sCMOS camera attached to a Nikon TI inverted epi-fluorescent microscope.

### Assessment of sample purity and polar capsule integrity

Polar capsules and remaining valves were enumerated using a counting chamber (BOECO Germany), in each post-isolation sample. Only samples that contained > 90% polar capsules (<10% valves) were used further. Polar capsule structural integrity was assessed visually both unstained and stained with Toluidine blue 0.01%. Only samples containing >85% intact capsules were analyzed further.

### Proteomic sample preparation and analysis

Three biological samples of isolated polar capsules harvested from different batched of lab-infected fish were subjected to tandem mass spectrometry (MS\MS) analysis at the Smoler Protein Research Center at the Technion, Israel. Each sample contained approximately one million polar capsules >90% purity, >85% intact). Samples were sonicated for 5 min in L&R Ultrasonic Quantrex 280 Cleaner and dissolved in standard 1% SDS PAGE protein sample buffer, with two heating cycles of 95° for 5 min. The samples were separated on a 7.5% SDS PAGE (Biorad) gel for 12 min at 120 V, then stained using Imperial™ Protein Stain (ThermoFisher), per the manufacturer’s instructions. The proteins in the gel were reduced with 3 mM DTT (60 °C for 30 min), modified with 10 mM iodoacetamide in 100 mM ammonium bicarbonate (in the dark at room temperature for 30 min) and digested in 10% acetonitrile and 10 mM ammonium bicarbonate with modified trypsin (Promega) at a 1:10 enzyme-to-substrate ratio (overnight at 37 °C). The resultant peptides were desalted using C18 tips and analyzed by LC-MS/MS. The peptides were resolved by reverse-phase chromatography on 0.075 × 180-mm fused silica capillaries (J&W) packed with Reprosil reversed-phase material (Dr. Maisch GmbH, Germany). Peptides were eluted using a linear 60 min gradient of 5% to 28%, 15 min gradient of 28% to 95% and 15 min at 95% acetonitrile with 0.1% formic acid in water at 0.15 μl/min. Mass spectrometry was performed by a Q Exactive plus mass spectrometer (Thermo) in a positive mode using data-dependent acquisition of the top-10 precursors, fragmented by collision induces dissociation (HCD).

Mass spectrometry data was analyzed using Byonic (Protein Metrics) software against the Open Reading Frames (ORFs) of our reference *C. shasta* transcriptome, generated using TransDecoder (https://transdecoder.github.io/), set at a minimum length of 30 amino acids. A full description of the assembly and annotation of the *C. shasta* transcriptome is presented elsewhere. A search was run with the following parameters: mass tolerances of 10 ppm for MS1 and 20 ppm for MS2, allowing 2 missed cleavages for trypsin digestions, with allowed modifications: fixed carbamidomethylation of C, maximum of three common modifications including de-amidation (N,Q) and oxidation (M, P) and one rare modification of C-terminal amidation. Peptide FDR was set to 1%^62^. Unidentified spectra were exported as MGF and re-searched with a mass window (“wildcard search”) of 350 Da (−100 Da to +250 Da).

The protein identification cutoff was set to at least two spectra containing one unique peptide. Hits in only one biological sample were dismissed. ORFs were annotated using Blast2GO (4.0.7) (default settings –5.2.2017), with BLAST + (2.3.0) used for Local blast (E val - 1E^−5^) and basic FASTA manipulations. Reciprocal Best BLAST Hits^63^ (protein vs. protein database) were done against the myxozoan *Thelohanellus kitauei* proteins^42^. Sequence alignments were conducted using CLC sequence viewer 7.7.1 (Aarhus, Denmark). The identified protein InterPro (IPR) domains of *C. shasta* polar capsules was compared to the IPR domains of nematocysts of jellyfish, sea anemone and *Hydra* ^34^ using known toxin and carbohydrate-related domains. The number of IPR domains was normalized to the total IPR domains found in each organism’s capsules. The protein sequences, Gene Ontology (GO) annotations and IPR results for identified proteins, and results of the Byonic searches are shown in Supplementary Table S1.

### Phylogenetic analyses

*C. shasta* nematogalectin and minicollagen sequences were aligned with published myxozoan sequences from NCBI using CLC sequence viewer software version 7.7.1 (Aarhus, Denmark). Multiple sequence alignments were edited manually to remove low confidence regions before progressing to tree construction. Phylogenetic neighbor joining (NJ) trees were created using the Jukes-Cantor model with 10,000 bootstrap replicates using CLC.

## Electronic supplementary material

Video 1

Table S1

Table S2

Supplementary information

## References

[1] Jimenez-Guri E, Philippe H, Okamura B, Holland PWH (2007). *Buddenbrockia* is a cnidarian worm. Science.

[2] Holland JW, Okamura B, Hartikainen H, Secombes CJ (2010). A novel minicollagen gene links cnidarians and myxozoans. Proc. R. Soc. B.

[3] Nesnidal MP, Helmkampf M, Bruchhaus I, El-Matbouli M, Hausdorf B (2013). Agent of whirling disease meets orphan worm: phylogenomic analyses firmly place Myxozoa in Cnidaria. PLoS ONE.

[4] Feng J-M (2014). New phylogenomic and comparative analyses provide corroborating evidence that Myxozoa is Cnidaria. Mol Phylogenet Evol.

[5] Foox J, Siddall ME (2015). The road to Cnidaria: history of phylogeny of the Myxozoa. J Parasitol.

[6] Chang ES (2015). Genomic insights into the evolutionary origin of Myxozoa within Cnidaria. Proc. Natl Acad. Sci. USA.

[7] Yahalomi, D. *et al*. The multipartite mitochondrial genome of Enteromyxum leei (Myxozoa): eight fast evolving megacircles. *Mol Biol Evol* (2017). 10.1093/molbev/msx072 28333349

[8] Okamura, B., Gruhl, A. & Bartholomew, J. L. *Myxozoan evolution, ecology and development*. (Springer, 2015).

[9] Lom J, Dyková I (2006). Myxozoan genera: definition and notes on taxonomy, life-cycle terminology and pathogenic species. Folia Parasitol..

[10] Canning EU, Okamura B (2003). Biodiversity and evolution of the Myxozoa. Adv Parasitol.

[11] Bartholomew JL, Whipple MJ, Stevens DG, Fryer JL (1997). The life cycle of *Ceratomyxa shasta*, a myxosporean parasite of Salmonids, requires a freshwater Polychaete as an alternate host. J Parasitol.

[12] Okamura B, Gruhl A (2016). Myxozoa and *Polypodium*: A common route to endoparasitism. Trends in Parasitology.

[13] Shpirer E (2014). Diversity and evolution of myxozoan minicollagens and nematogalectins. BMC Evol Biol.

[14] Reft, A. J. & Daly, M. Morphology, distribution, and evolution of apical structure of nematocysts in hexacorallia. *J Morphol*, n/a-n/a, doi:10.1002/jmor.11014 (2011). 10.1002/jmor.11014 21960117

[15] Weill R (1938). L’interpretation des Cnidosporidies et la valeur taxonomique de leur cnidome. Leur cycle compare´ a‘ la phase larvaire des Narcome’duses cuninides. Travaux de la Station Zoologique de Wimereaux.

[16] Tardent P (1995). The cnidarian cnidocyte, a hightech cellular weaponry. Bioessays.

[17] Beckmann A, Özbek S (2012). The nematocyst: a molecular map of the cnidarian stinging organelle. Int J Dev Biol.

[18] Nüchter T, Benoit M, Engel U, Ozbek S, Holstein TW (2006). Nanosecond-scale kinetics of nematocyst discharge. Curr Biol.

[19] Holstein T, Tardent P (1984). An ultrahigh-speed analysis of exocytosis: nematocyst discharge. Science.

[20] Weber J (1990). Poly(gamma-glutamic acid)s are the major constituents of nematocysts in *Hydra* (Hydrozoa, Cnidaria). J Biol Chem.

[21] Szczepanek S, Cikala M, David CN (2002). Poly-γ-glutamate synthesis during formation of nematocyst capsules in. Hydra. J Cell Sci.

[22] Ayalon A, Shichor I, Tal Y, Lotan T (2011). Immediate topical drug delivery by natural submicron injectors. Int. J. Pharm..

[23] Park S (2017). The nematocyst’s sting is driven by the tubule moving front. J R Soc Interface.

[24] Cannon Q, Wagner E (2003). Comparison of discharge mechanisms of Cnidarian cnidae and Myxozoan polar capsules. Rev Fish Sci.

[25] Kallert DM, Ponader S, Eszterbauer E, El-Marbouli M, Haas W (2007). Myxozoan transmission via actinospores: new insights into mechanisms and adaptations for host invasion. Parasitology.

[26] Ben-David J (2016). Myxozoan polar tubules display structural and functional variation. Parasites & Vectors.

[27] Balasubramanian PG (2012). The proteome of *Hydra* nematocyst. J Biol Chem.

[28] Brinkman DL (2012). Venom proteome of the Box Jellyfish *Chironex fleckeri*. PLoS ONE.

[29] Moran Y (2013). Analysis of soluble protein contents from the nematocysts of a model sea anemone sheds light on venom evolution. Mar Biotechnol.

[30] Weston AJ (2013). Proteomic characterisation of toxins isolated from nematocysts of the South Atlantic jellyfish *Olindias sambaquiensis*. Toxicon.

[31] Li, R. *et al*. Jellyfish venomics and venom gland transcriptomics analysis of *Stomolophus meleagris* to reveal the toxins associated with sting. *J Proteomics* (2014). 10.1016/j.jprot.2014.04.011 24747124

[32] Li R (2016). Combined proteomics and transcriptomics identifies sting-related toxins of jellyfish Cyanea nozakii. Journal of Proteomics.

[33] Ponce D, Brinkman D, Potriquet J, Mulvenna J (2016). Tentacle transcriptome and venom proteome of the Pacific Sea Nettle, *Chrysaora fuscescens* (Cnidaria: Scyphozoa). Toxins.

[34] Rachamim T (2015). The dynamically evolving nematocyst content of an Anthozoan, a Scyphozoan, and a Hydrozoan. Mol Biol Evol.

[35] Hoffmaster J, Sanders J, Rohovec J, Fryer J, Stevens D (1988). Geographic distribution of the myxosporean parasite, *Ceratomyxa shasta* Noble, 1950, in the Columbia River basin, USA. Journal of Fish Diseases.

[36] Ray RA, Holt RA, Bartholomew JL (2012). Relationship between temperature and *Ceratomyxa shasta*–induced mortality in Klamath River salmonids. Journal of Parasitology.

[37] Hallett SL (2012). Density of the waterborne parasite *Ceratomyxa shasta* and its biological effects on salmon. Appl Environ Microbiol.

[38] Hallett, S. L. & Bartholomew, J. L. in *F*ish parasit*es*: pat*hobiology and protection* (eds P.T.K. Woo & K. Buckmann) (Wallingford: CAB International, 2011).

[39] Qian C (2014). Dielectrophoresis for bioparticle manipulation. International Journal of Molecular Sciences.

[40] Lotan A, Fishman L, Loya Y, Zlotkin E (1995). Delivery of a nematocyst toxin. Nature.

[41] Shaoul E, Ayalon A, Tal Y, Lotan T (2012). Transdermal delivery of scopolamine by natural submicron injectors: *in-vivo* study in pig. PLoS ONE.

[42] Yang Y (2014). The genome of the Myxosporean *Thelohanellus kitauei* shows adaptations to nutrient acquisition within its fish host. Genome Biol Evol.

[43] Milde S (2009). Characterization of taxonomically restricted genes in a phylum-restricted cell type. Genome Biol.

[44] Gibbs GM, Roelants K, O’Bryan MK (2008). The CAP superfamily: Cysteine-rich secretory proteins, antigen 5, and pathogenesis-related 1 proteins—roles in reproduction, cancer, and immune defense. Endocr. Rev..

[45] Kodádková A, Bartošová-Sojková P, Holzer AS, Fiala I (2015). *Bipteria vetusta* n. sp. – an old parasite in an old host: tracing the origin of myxosporean parasitism in vertebrates. Int J Parasitol.

[46] Cheng I-F, Chang H-C, Hou D, Chang H-C (2007). An integrated dielectrophoretic chip for continuous bioparticle filtering, focusing, sorting, trapping, and detecting. Biomicrofluidics.

[47] Rozitsky L (2013). Quantifying continuous-flow dielectrophoretic trapping of cells and micro-particles on micro-electrode array. Biomed. Microdevices.

[48] Zhang C, Khoshmanesh K, Mitchell A, Kalantar-zadeh K (2010). Dielectrophoresis for manipulation of micro/nano particles in microfluidic systems. Anal. Bioanal. Chem..

[49] Park S, Capelin D, Piriatinskiy G, Lotan T, Yossifon G (2017). Dielectrophoretic characterization and isolation of jellyfish stinging capsules. Electrophoresis.

[50] Hwang JS (2010). Nematogalectin, a nematocyst protein with GlyXY and galectin domains, demonstrates nematocyte-specific alternative splicing in Hydra. PNAS 2010 107 (43)..

[51] Adamczyk P (2010). A non-sulfated chondroitin stabilizes membrane tubulation in cnidarian organelles. J Biol Chem.

[52] Yamada S, Morimoto H, Fujisawa T, Sugahara K (2007). Glycosaminoglycans in *Hydra magnipapillata* (Hydrozoa, Cnidaria): demonstration of chondroitin in the developing nematocyst, the sting organelle, and structural characterization of glycosaminoglycans. Glycobiology.

[53] Denker E, Bapteste E, Le Guyader H, Manuel M, Rabet N (2008). Horizontal gene transfer and the evolution of cnidarian stinging cells. Curr Biol.

[54] Hwang JS (2007). The evolutionary emergence of cell type-specific genes inferred from the gene expression analysis of Hydra. Proc Natl Acad of Sci USA.

[55] Mariottini GL (2014). Hemolytic venoms from marine cnidarian jellyfish–an overview. J Venom Res.

[56] Jouiaei M (2015). Ancient venom systems: A review on Cnidaria toxins. Toxins.

[57] Foox J, Ringuette M, Desser SS, Siddall ME (2015). In silico hybridization enables transcriptomic illumination of the nature and evolution of Myxozoa. BMC Genomics.

[58] Bartholomew J, Smith C, Rohovec J, Fryer J (1989). Characterization of a host response to the myxosporean parasite, *Ceratomyxa shasta* (Noble), by histology, scanning electron microscopy and immunological techniques. J Fish Dis.

[59] Koifman, N. a., Biran, I., Aharon, A., Brenner, B. & Talmon, Y. A direct-imaging cryo-EM study of shedding extracellular vesicles from leukemic monocytes. *J Struct Biol* (2017). 10.1016/j.jsb.2017.02.004 28254382

[60] Issman L, Talmon Y (2012). Cryo‐SEM specimen preparation under controlled temperature and concentration conditions. JMic.

[61] Gagnon ZR (2011). Cellular dielectrophoresis: applications to the characterization, manipulation, separation and patterning of cells. Electrophoresis.

[62] Bern MW, Kil YJ (2011). Two-dimensional target decoy strategy for shotgun proteomics. J Proteome Res.

[63] Altenhoff AM, Dessimoz C (2009). Phylogenetic and functional assessment of orthologs inference projects and methods. PLoS Comput Biol.

