# Supplementary material for: Functional and proteomic analysis of Ceratonova shasta (Cnidaria: Myxozoa) polar capsules reveals adaptations to parasitism
Source: Sci Rep. 2017 Aug 21;7:9010. doi: 10.1038/s41598-017-09955-y (PMC5566210; doi:10.1038/s41598-017-09955-y)
Supplement: Supplementary file 4 — Supplementary information [file 41598_2017_9955_MOESM4_ESM.pdf]

Functional and proteomic analysis of *Ceratonova shasta* (Cnidaria: Myxozoa) polar capsules reveals adaptations to parasitism

Gadi Piriatinskiy[1], Stephen D. Atkinson[2], Sinwook Park[3], David Morgenstern[1], Vera Brekhman[1], Gilad Yossifon[3], Jerri L. Bartholomew[2] and Tamar Lotan[1*]

Supplementary Information:

Figure S1. *C. shasta* nematogalectins phylogenetic tree.

Figure S2. *C. shasta* minicollagen phylogenetic tree

Table S1. (excel file) List of proteins identified in the study including annotation, proteomic data and sequences

Table S2. (excel file) InterPro domain analysis of *C. shasta*, Anemonia, Aurelia and Hydra

Video 1. Polar capsules separation process on the DEP-chip platform

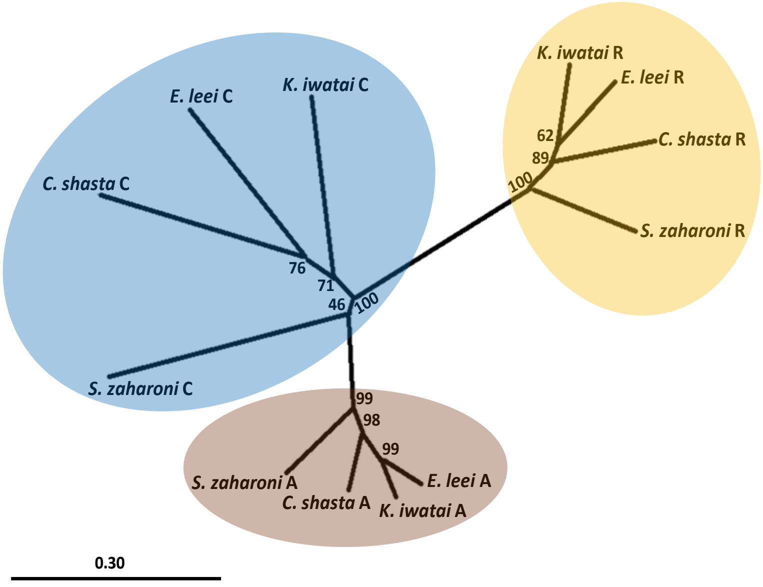

Figure S1. *C. shasta* nematogalectins phylogenetic tree. Neighbor-joining phylogenetic tree based on myxozoan nematogalectin types A, C and R (Related) sequences. Bootstrap = 10K. NCBI accession numbers: *E. leei*: nematogalectin A - CDU32397, nematogalectin C - CDU32398, nematogalectin R - CDU32399; *K. iwatai*: nematogalectin A - CDU32395, nematogalectin C - CDU32394, nematogalectin R - CDU32396; *S. zaharoni*: nematogalectin A - CDU32392, nematogalectin C - CDU32393, nematogalectin R - CDU32391.

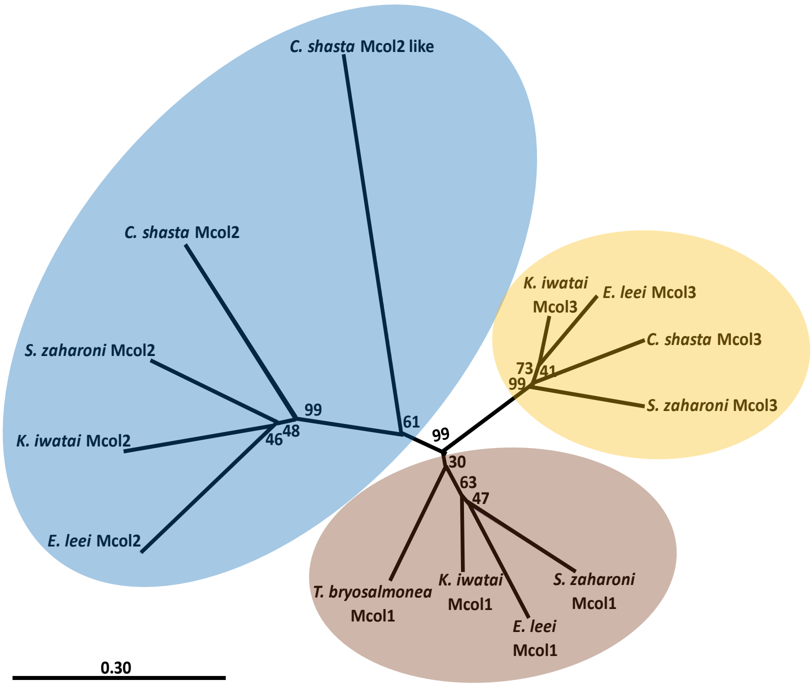

Figure S2. ***C. shasta* minicollagen phylogenetic tree.** Neighbor joining phylogenetic tree based on myxozoan minicollagens (Mcol) types 1, 2 and 3. Bootstrap = 10K. NCBI accession numbers: *E. leei*: Mcol1 - CDU32385, Mcol2 - CDU32386, Mcol3 - CDU32387; *K. iwatai*: Mcol1 - CDU32382, Mcol2 - CDU32383, Mcol3 - CDU32384; *S. zaharoni*: Mcol1 - CDU32388, Mcol2 - CDU3239, Mcol3 - CDU32390; *T. bryosalmonae* Mcol1 - CBJ19443.
